# Mathematical Modeling of Risk-Taking in Bipolar Disorder: Evidence of Reduced Behavioral Consistency, With Altered Loss Aversion Specific to Those With History of Substance Use Disorder

**DOI:** 10.5334/cpsy.61

**Published:** 2022-05-24

**Authors:** Carly A. Lasagna, Timothy J. Pleskac, Cynthia Z. Burton, Melvin G. McInnis, Stephan F. Taylor, Ivy F. Tso

**Affiliations:** 1 Department of Psychology, University of Michigan, Ann Arbor, Michigan, US; 2 Department of Psychiatry, University of Michigan, Ann Arbor, Michigan, US; 3 Department of Psychology, University of Kansas, Lawrence, Kansas, US

**Keywords:** Bipolar disorder, Substance use, Risk-taking, Risk behavior, Reward processing, Executive function, Computational model, loss aversion, behavioral consistency

## Abstract

Bipolar disorder (BD) is associated with excessive pleasure-seeking risk-taking behaviors that often characterize its clinical presentation. However, the mechanisms of risk-taking behavior are not well-understood in BD. Recent data suggest prior substance use disorder (SUD) in BD may represent certain trait-level vulnerabilities for risky behavior. This study examined the mechanisms of risk-taking and the role of SUD in BD via mathematical modeling of behavior on the Balloon Analogue Risk Task (BART). Three groups—18 euthymic BD with prior SUD (BD+), 15 euthymic BD without prior SUD (BD–), and 33 healthy comparisons (HC)—completed the BART. We modeled behavior using four competing hierarchical Bayesian models, and model comparison results favored the Exponential-Weight Mean-Variance (EWMV) model, which encompasses and delineates five cognitive components of risk-taking: prior belief, learning rate, risk preference, loss aversion, and behavioral consistency. Both BD groups, regardless of SUD history, showed lower behavioral consistency than HC. BD+ exhibited more pessimistic prior beliefs (relative to BD– and HC) and reduced loss aversion (relative to HC) during risk-taking on the BART. Traditional measures of risk-taking on the BART (adjusted pumps, total points, total pops) detected no group differences. These findings suggest that reduced behavioral consistency is a crucial feature of risky decision-making in BD and that SUD history in BD may signal additional trait vulnerabilities for risky behavior even when mood symptoms and substance use are in remission. This study also underscores the value of using mathematical modeling to understand behavior in research on complex disorders like BD.

Risk-taking is a central and defining feature of bipolar disorder (BD), as its clinical presentation often includes pleasure- or reward-seeking pursuits despite the potential for negative consequences. Such behaviors include greater involvement in reckless and intoxicated driving, excessive spending, and impulsive aggression (Perroud et al., 2011; Reinharth et al., 2017), but the most prevalent of these is substance use, with an estimated 61% of individuals with BD experiencing a lifetime comorbid substance use disorder (SUD; Cerullo & Strakowski, 2007). Although risky behaviors in BD likely have multiple etiologies, abnormalities in reward processing and impaired cognitive control are potential driving factors. However, the underlying mechanisms by which such factors determine risk-taking behavior in BD are not well understood. The present study aimed to elucidate the cognitive processes underlying risk-taking behavior in BD using a mathematical modeling approach.

## Introduction

### Processes underlying risk-taking behavior

The act of risk-taking draws on a constellation of underlying processes that interact during the decision-making process (Pleskac, 2015). It is generally understood (see Ahn et al., 2017; Pleskac, 2008; Yechiam et al., 2005 for reviews) that these consist of: (1) motivational processes that shape how rewards are evaluated and the desire to pursue a reward (Gray, 1981; Kim & Lee, 2011; Suhr & Tsanadis, 2007; Zuckerman, 1979) or avoid consequences (Gray, 1981); (2) cognitive control processes that enable people to appropriately modulate these drives and prepotent response styles, adapting and inhibiting behavior to achieve goals (Diamond, 2013), (3) prior expectations or ‘mental models’ that people have about the outside world, and (4) learning process that control how people learn from experience and update their prior expectations about the world. Alterations to any of these underlying processes can lead to behavior that is overly risk-seeking (Ball et al., 1994; Hoyle et al., 2000; Jonah, 1997), where pleasurable experience is pursued despite the potential of harm. For example, abnormally high motivation toward reward may increase tolerance of risk, altering behavioral tendencies and manifesting as risk-seeking behavior. Alternatively, a diminished ability to learn from experiences can result in individuals continuing to choose convenient or seemingly attractive options that produce losses (Busemeyer & Stout, 2002). Finally, executive function impairments may lead to a failure to adapt behavior to situational demands, making more erratic decisions in a way that is also (inadvertently) risk-seeking. Thus, there are various avenues to risk-taking behavior.

### Risk-taking behavior in BD

Considering these different pathways to risk-taking, we face a unique problem in trying to isolate the source of risky behavior in BD: individuals with BD show chronic abnormalities in several of these underlying processes. Motivational and cognitive aberrations are common across mood states, as indicated by self-reports of higher reward sensitivity (Meyer et al., 2001; Salavert et al., 2007), sensation seeking (Cronin & Zuckerman, 1992) and an impaired ability to inhibit inappropriate responses (Martinez-Aran et al., 2004; Mur et al., 2007; Ryan et al., 2012). These differences would suggest that individuals with BD are more prone to risk-taking regardless of mood state. Yet, factors that may offset these vulnerabilities are also present in BD. For example, individuals with BD show greater aversion to negative consequences across mood states as indicated by self-reports (Meyer et al., 2001; Yechiam et al., 2008). It is possible that this would be especially evident during euthymia, exacerbated by the salience of negative consequences experienced from risky behavior in past manic episodes (which may also shape risk preferences and loss aversion over time). Limited by several methodological issues, the current literature does not offer a precise understanding of how these processes (i.e., motivation, prior experience, cognition, loss aversion) work together to result in risky behavior in BD. Below we highlight and discuss two specific issues that we attempted to address in the current study.

#### Inconsistent behavioral findings due to sample heterogeneity

In both clinical and non-clinical contexts, risk-taking behavior has been most often studied using controlled laboratory tasks theorized to tap similar processes as real-world risk-taking behaviors. Behavioral findings using this approach have been inconsistent in BD and difficult to compare across studies (Bauer et al., 2017; Hidiroǧlu et al., 2013; Holmes et al., 2009; Linke et al., 2013; Ramírez-Martín et al., 2020; Reddy et al., 2014; Scholz et al., 2016). One reason they have been difficult to compare is the differences in mood state between investigations (Ramírez-Martín et al., 2020). Some have studied purely euthymic samples (Hidiroǧlu et al., 2013; Linke et al., 2013; Scholz et al., 2016), while others have studied samples containing a mixture of hypo/manic, euthymic, and depressed participants (Bauer et al., 2017; Holmes et al., 2009; Reddy et al., 2014). Additionally, studies often collapse together BD groups with and without prior SUD (Hidiroǧlu et al., 2013; Linke et al., 2013; Reddy et al., 2014; Scholz et al., 2016) and recent evidence suggests that motivational abnormalities related to risk-taking in BD are driven by subgroups of BD with prior SUD (Henry et al., 2001; Holmes et al., 2009). Without considering SUD history, it is unclear whether abnormal risk-taking behavior is an endophenotype of BD, or of a subtype with trait vulnerabilities to risky behavior, including substance use (Frey et al., 2017).

#### Traditional measurements are insensitive to underlying mechanisms

Traditionally, laboratory behavioral studies index risk-taking with a single metric based on overall performance on gambling tasks, such as the ‘average number of pumps on unpopped balloons’ on the Balloon Analogue Risk Task (BART; Lejuez et al., 2002) or the ‘number of cards’ on the Columbia Card Task (Figner et al., 2009). These metrics indicate the mean magnitude of risk behavior across trials but provide limited insight regarding the underlying cognitive processes. In BD, many underlying vulnerabilities could contribute to risk-taking behavior. For example, ‘heightened risk-taking’ can arise from high-risk preference, poor response inhibition, and/or low loss aversion, where alterations in all of these processes have been found in BD (Chandler et al., 2009; Holmes et al., 2009; Swann et al., 2003). Therefore, an accurate explanation of abnormal risk-taking behaviors in BD requires methods that can uncover the latent mechanisms.

### Computational modeling as an alternative

Computational models of risk-taking behavior have been proposed as solutions to the limitations of traditional behavioral metrics. They enable researchers to extract indicators of the underlying mechanisms of behavior from theory-linked equations and subject them to direct hypothesis testing. With computational modeling, we go from being able to identify the presence of an abnormality, to being able to explain the underlying processes it arose from. They provide a formal mechanistic understanding of behavior (Huys et al., 2016) that is capable of uncovering abnormalities that traditional measures are not sensitive to detect (Silverstein et al., 2017; Timmer et al., 2017; Vassileva et al., 2013; Yechiam et al., 2008; Zorowitz et al., 2020). In BD research, however, very few have modeled risk-related behavior in experimental contexts. Foundational work comes from Yechiam and colleagues (Yechiam et al., 2008), who modeled impulsive decision-making in BD during the Iowa Gambling Task (Bechara et al., 1994) using the Expectancy Valence Model (Busemeyer & Stout, 2002; Yechiam et al., 2008). They showed that manic-BD (relative to euthymic-BD and HC) were more inconsistent in choosing the higher-valued option. This suggests that a failure to consistently adhere to a responding schema that would maximize long-term reward is likely one factor contributing to altered risk-taking in BD. Other contributing psychological processes remain to be delineated in order to gain a full understanding of what drives excessive involvement in pleasurable activities in BD (American Psychiatric Association, 2013).

### The present study

To probe risk-taking in BD, the present study examined behavior on the Balloon Analogue Risk Taking (BART; Lejuez et al., 2002) in three groups: euthymic BD with prior SUD (BD+), euthymic BD without prior SUD (BD–), and healthy comparisons (HC). The BART is a computerized task devised by Lejuez and colleagues (2002) to measure clinically relevant risky behavior, and performance has been shown to predict real-world risk-taking including substance use (Lejuez et al., 2003) and risky sexual behavior (Lejuez et al., 2004; see Lauriola et al., 2014 for meta-analysis).

During the BART, participants earn rewards by sequentially inflating virtual balloons. On any balloon, each successful pump adds to the total reward the participant can earn on the current trial. For example, at 10 points/pump, after 10 successful pumps the current balloon is worth 100 points, after 50 it is worth 5000 points, and so on. However, not all pumps are successful—if the participant pumps and the balloon bursts, they earn nothing for that balloon. Thus, pumps made on a given balloon represent a series of choices between two alternatives: (1) keep the total reward amount they have accumulated, or (2) pump the balloon and risk losing the amount accumulated in pursuit of greater potential reward. This sequential format has ecological validity because many risk-taking behaviors in the real world occur in a similar sequential manner (e.g., gambling, repeated use of illicit substances). From a theoretical perspective, behavior on the first balloon is likely determined by several processes: prior belief, risk preferences/tendencies, aversion to loss, and decision-making patterns (i.e., consistent vs erratic decision strategy). Then, as the decision-maker gains experience with the task, on subsequent balloons, we expect them to learn from experience and incorporate this into their behavior on subsequent trials.

Several computational models have been posed to characterize the cognitive processes underlying risk-taking on the BART (Park et al., 2021; Pleskac, 2008; van Ravenzwaaij et al., 2011; Wallsten et al., 2005) (for discussion see Park et al., 2021). Here, we focus on the novel Exponential-Weight Mean-Variance (EWMV) model (Park et al., 2021), which models five constituent cognitive components of risk-taking behavior: prior belief, learning rate, risk preference, behavioral consistency, and loss aversion. We chose the EWMV model because it shows good parameter recovery, outperformed existing BART models (Park et al., 2021; Wallsten et al., 2005), and its parameters index various processes likely involved in risk-taking in euthymic BD (namely, risk preference, behavioral consistency, and loss aversion). Furthermore, in our current study, we subjected the EWMV to a model competition comparing its performance against several other models using leave-one-out cross validation. The EWMV equaled or outperformed the other models.

Using the EWMV we (1) assessed group differences at the mechanistic level, and (2) explored relationships between parameters and external self-report and neuropsychological measures to support the validity of those parameters. We anticipated the risk preference and loss aversion parameters of the EWMV model would capture motivational aspects of risk-taking (i.e., motivation towards reward) and that the behavioral consistency and learning rate parameters would reflect cognitive control aspects of risk-taking. As such, we expected that risk preference and loss aversion parameters would correlate with self-reported motivational measures (sensation seeking, behavioral activation/inhibition), and that behavioral consistency and learning rate parameters would correlate with executive functioning performance on neuropsychological tests. Because of previous reports of heightened self-reported reward sensitivity (Meyer et al., 2001; Salavert et al., 2007) and sensation seeking (Cronin & Zuckerman, 1992), as well as poorer executive functioning, in BD across mood episodes (Martinez-Aran et al., 2004; Mur et al., 2007; Ryan et al., 2012), we hypothesized that BD would show, as indicated by model parameters, increased risk preference, reduced loss aversion, lower behavioral consistency, and lower learning rates than HC. Previous studies have also shown that, among populations with BD, more pronounced cognitive impairments (Levy et al., 2008; van Gorp et al., 1998) and higher self-reported reward sensitivity (Alloy et al., 2009) are found in those who have engaged in problematic substance use. Moreover, BD+ has been found to have greater levels of impulsivity than BD– (Swann et al., 2005). Thus, we also hypothesized in the domain of risk-taking that BD+ would exhibit more pronounced cognitive/motivational differences than BD– would be more pronounced among relative to BD–.

## Methods

### Participants

Participants were 33 individuals with BD (currently euthymic) and 32 HC. These participants were involved in a parent fMRI study, as such the sample sizes were determined by a power analysis for fMRI effect sizes (not related to the expected behavioral effect sizes of the BART). All were ages 18–55 without history of medical conditions with neurological sequelae. BD met criteria for either bipolar disorder I, II, or not otherwise specified (NOS) according to the Structured Clinical Interview for DSM-IV-TR (First et al., 2002) (SCID-IV), and euthymic state was confirmed via clinician ratings (i.e., <10 on the Hamilton Depression Rating Scale [HAM-D; Hamilton, 1960] and <7 on the Young Mania Rating Scale [YMRS; Young et al., 1978]). Within BD, 18 met SCID-IV criteria for past substance abuse/dependence (BD+) and 15 did not (BD–). HC who had lifetime axis-I disorders (according to SCID-IV) or immediate family with bipolar/psychotic disorder(s) were excluded from the study.

### Procedure

This study was approved by the Institutional Review Board at the University of Michigan Medical School. All participants provided informed consent and were compensated for their time. Testing was completed in a single session in the following order: SCID and HAM-D/YMRS; BART; neuropsychological testing and self-reports.

### Materials

#### BART

**Figure 1:**
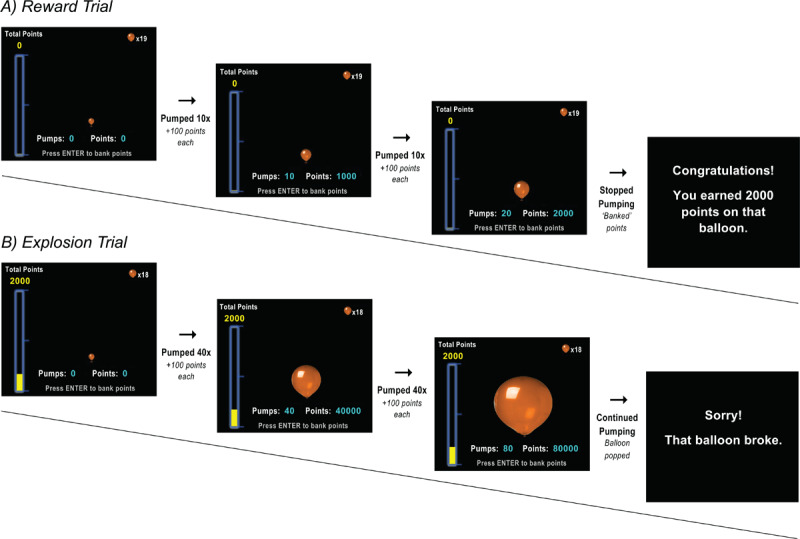
Sample balloons for the computerized Balloon Analogue Risk Task (BART) used in the present study. Participants earned 100 points to a temporary account with each pump made (balance at bottom of screen) and ‘banked’ points were saved to a permanent account (balance at top of screen). Balloons were programmed to burst at unknown breakpoints. **A)** Example of trial in which the participant successfully banked points and earned a reward. **B)** Example of trial in which the balloon burst.

The BART was programmed in E-Prime 2.0 Standard (PST, Inc., Pittsburgh, PA) and consisted of 6 practice and 20 experimental balloons. An example of the task presentation is displayed in Figure 1. During the BART, a simulated balloon is shown on a computer screen as represented by an image of the balloon. Pressing the “Space” bar pumped the balloon increasing the diameter of the balloon in all directions (about 2–3 mm). If the balloon did not burst, then the participant earned 100 points per pump. If the balloon did burst, then the balloon image disappeared and a message told the participant the balloon broke (Figure 1B). If the balloon broke, then the participant lost all the points they earned for that balloon. Thus, the participant must decide when to stop pumping and collect the points earned on that balloon. Participants were deliberately given only general information about when balloons may burst (i.e., “Each balloon can pop anywhere from the first pump all the way through enough pumps to make the balloon fill the screen”). To stop and collect the points, participants had to hit “Enter.” Doing so ended the balloon trial and transferred their earned points to a bank; the balloon image disappeared and a message told the participant total points they had earned for that balloon (Figure 1A). The explosion point for each balloon was pseudorandomized over practice and experimental trials such that burst thresholds were approximately normally distributed between 1 and 128 pumps with a mean of 64 (practice breakpoints: 57, 100, 19, 93, 67, 48 [M = 64, SD = 30]; experimental breakpoints: 110, 23, 121, 94, 59, 102, 47, 16, 74, 62, 65, 56, 92, 45, 82, 35, 76, 27, 84, 10 [M = 64, SD = 31]). This process means that the probability that the balloon would burst on pump i given the balloon given the balloon had not burst on i–1 trials is: 1/(128 – i +1). We used the same explosion points and sequence of breakpoints across participants.

During experimental trials, each pump earned 100 points to a temporary account. Points banked during the task were added to participants’ compensation at a rate of 1¢ per 100 points. Practice trials were identical in appearance to experimental trials, but participants were informed that these were not part of the actual experimental task. This helped ensure that risk-taking behavior on experimental trials was as stable and did not reflect acclimation to the experiment. Behavior during practice was qualitatively different from experimental trials (because no risk or reward was involved during practice), so practice responses were omitted from analyses. Standard BART measures—adjusted pumps (average pumps on unpopped balloons), total pops, and total points—were also computed for each participant to facilitate comparison with model parameters.

#### Self-report and neuropsychological measures

Complete descriptions for all self-report/neuropsychological measures are provided in Supplement 1.1 to 1.3. In summary, participants completed self-reports (Behavioral Inhibition/Activation System Scale [BIS/BAS; Carver & White, 1994], Sensation Seeking Scale [SSS; Zuckerman et al., 1964]) and a battery of five neurocognitive tests of executive functioning (see Supplement 1.3). A principal components analysis (PCA) was performed on standardized scores from these five neurocognitive tests to derive a single executive functioning component.

## Computational modeling

In the present paper we focus on the EWMV model to investigate the mechanisms of risk-taking behavior in BD. However, we did not simply assume that the EWMV model was the best-fitting model for our three samples. Rather, we tested four separate models using hierarchical Bayesian estimation: the EWMV model, the Bayesian sequential risk-taking model (Wallsten et al., 2005) (reparameterized by Park et al., 2021) and two simpler baseline models. First, we describe the specific computations and assumptions involved in each model (see ‘Model descriptions’). Next, we review the modeling approach (see ‘Model implementation’) and procedures used to verify the integrity of parameters/predictions and select a winning model (see ‘Model evaluation’).

### Model descriptions

The computations involved in each of the four models tested are described in detail in sections 1.1 to 1.4 below. This information is also summarized in Table 1.

**Table 1:** Overview of models tested.


MODEL	PARAMETERS	ESTIMATED VIA

**Exponential-Weight Mean-Variance (EWMV) Model** Park et al. (2021)	*ψ* = Prior belief of burst *ξ* = Learning rate *ρ* = Risk preference *τ* = Behavioral consistency *λ* = Loss aversion	Equation 1 Equation 1 Equation 2 Equation 3 Equation 2

**Bayesian Sequential Risk-Taking (BSR) Model** Park et al. (2021); Wallsten et al. (2005)	*φ* = Prior belief of success *η* = Learning rate *γ* = Risk propensity *τ* = Behavioral consistency	Equation 4 Equation 4 Equation 6 Equation 7

**3-Parameter No Learning (3par) Model** Park et al. (2021)	*θ* = Prior belief of success *γ* = Risk propensity *τ* = Behavioral consistency	Equation 8 Equation 8 Equation 9

**2-Parameter No Learning (2par) Model** Adapted – van Ravenzwaaij et al. (2011)	*γ* = Risk propensity *τ* = Behavioral consistency	Equation 10 Equation 9


#### Exponential-weight mean-variance model (EWMV)

The exponential-weight mean-variance (EWMV) model assumes that decision makers have a subjective probability ( \[
p_k^{burst}
\] ) that pumping the current balloon, *k*, will cause the balloon to burst. Psychologically, \[
p_k^{burst}
\] depends on the decision maker’s prior belief and learning processes (via which prior beliefs are updated as they gain experience on the BART; Park et al., 2021). Mathematically, this is represented in Equation 1:


1 \[
p_k^{burst} = e^{-\xi \sum \nolimits_{i = 0}^{k - 1} n_i^{pumps}} \psi + \left({1 - e^{-\xi \sum \nolimits_{i = 0}^{k - 1} n_i^{pumps}}} \right)\left({\frac{{\sum \nolimits_{i = 0}^{k - 1} \left({n_i^{pumps} - n_i^{success}} \right)}}{{\sum \nolimits_{i = 0}^{k - 1} n_i^{pumps}}}} \right)\ with\ 0 < \psi < 1, \xi > 0
\]

where, \[
p_k^{burst}
\] is the weighted average of the decision maker’s prior belief that pumping will burst the balloon (*ψ*) and the ‘observed burst probability’, which is simply a ratio of the number of bursts ( \[
\mathop \sum \nolimits_{i = 0}^{k - 1} \left({n_i^{pumps} - n_i^{success}} \right)
\] ) to the total pumps that the decision-maker has made at that point in the task ( \[
\mathop \sum \nolimits_{i = 0}^{k - 1} n_i^{pumps}
\] ). The weight given to the observed burst probability depends on the total amount of evidence accumulated. The weight given to accumulated evidence is determined by both the total evidence accumulated and the learning rate (*ξ*), which indicates how readily the decision-maker incorporates new evidence into their prior experience. We should note that in the original model development, Park and colleagues (2021) interpret *ξ* simply in terms of a learning rate, but the rate of updating may also reflect the certainty of one’s prior beliefs. Both *ψ* and *ξ* are free parameters estimated from the data. Higher values for the prior belief parameter *ψ* indicate more pessimistic prior beliefs (i.e., the balloon is more likely to burst) and higher values for the learning rate parameter *ξ* indicate a decision-maker that more readily incorporates new evidence into their prior beliefs. Higher values for *ξ* may also signal a decision-maker that has less certainty in their prior beliefs and more readily updates their expectations as a function of this uncertainty.

Next, the EWMV framework assumes that the probability of a decision-maker pumping/stopping on pump opportunity l for a given balloon k depends on their current subjective burst probability ( \[
p_k^{burst}
\] ; from Equation 1) and their current subjective utilities for pumping ( \[
U_{kl}^{pump}
\] ) or stopping ( \[
U_{kl}^{stop}
\] ) at each pump opportunity l on any given balloon k. These notions of subjective utilities borrow from the principles of Prospect Theory (Kahneman & Tversky, 1979). The subjective utility of stopping ( \[
U_{kl}^{stop}
\] ) is fixed at 0 because stopping does not add any further reward to the total amount for the current balloon. The subjective utility of pumping ( \[
U_{kl}^{pump}
\] ), on the other hand, is determined by the perceived probability that pumping will cause the balloon to burst ( \[
p_k^{burst}
\] ), the amount of reward per successful pump, the decision-maker’s risk-taking preferences, and their aversion to loss. Computationally, this is captured in Equation 2:


2 \[
U_{kl}^{pump} = \left({1 - p_k^{burst}} \right)r - p_k^{burst}\lambda \left({l - 1} \right)r + \rho p_k^{burst}\left({1 - p_k^{burst}} \right){\left\{ {r + \lambda \left({l - 1} \right)r} \right\}^2}\ with\ \lambda > 0
\]

where, *r* is the amount of reward per pump, *ρ* is the decision-maker’s risk preference, and *λ* is their level of loss aversion. The value of *r* is constant and determined by the task design (e.g., 100 points in our implementation). Both *λ* and *ρ* are free parameters estimated from the data. Higher values for *ρ* indicate higher risk propensities and higher *λ* values indicate greater aversion to loss.

Finally, the probability of pumping on each opportunity *l* for a given balloon *k* is calculated using Equation 3:


3 \[
p_{kl}^{pump} = \frac{1}{{1 + {e^\tau }\left({U_{kl}^{stop} - U_{kl}^{pump}} \right)}}\ with\ \tau \ge 0
\]

where the likelihood of pumping ( \[
p_{kl}^{pump}
\] ) is determined by the value difference between subjective utilities of stopping ( \[
U_{kl}^{stop}
\] ) and pumping ( \[
U_{kl}^{pump}
\] ), and the decision-maker’s behavioral consistency (τ; i.e., inverse temperature) which is estimated from the data. Higher *τ* values indicate behavior that is more deterministic in terms of maximizing their subjective utility, while lower values indicate behavior that changes more erratically from trial-to-trial.

In sum, the EWMV model estimates five variables of interest: prior belief of burst (*ψ*), learning rate (*ξ*), risk preference (*ρ*), loss aversion (*λ*), and behavioral consistency (*τ*).

#### Bayesian sequential risk-taking model (BSR)

The BSR model assumes that the decision-maker has an initial subjective value that pumping will burst the balloon, which is updated after each balloon based on prior experience (Pleskac, 2008; Wallsten et al., 2005). Initial belief undergoes updating as evidence is accumulated, producing the decision-maker’s subjective probability that the balloon will burst ( \[
p_k^{burst}
\] ) given by Equation 4:


4 \[
p_k^{burst} = 1 - \frac{{\phi + \eta \mathop \sum \nolimits_{i = 0}^{k - 1} n_i^{succ}}}{{1 + \eta \mathop \sum \nolimits_{i = 0}^{k - 1} n_i^{pumps}}}\,with\;0 < \phi < 1,\eta > 0
\]

where *φ* is the initial belief that pumping will not make the balloon burst (i.e., prior belief of success), *η* is the learning rate, and the observed probability of a successful pump is given by the ratio of successful pumps ( \[
\mathop \sum \nolimits_{i = 0}^{k - 1} n_i^{succ}
\] ) to total pumps ( \[
\mathop \sum \nolimits_{i = 0}^{k - 1} n_i^{pumps}
\] ). We used the reparameterized version of the BSR that improves the recoverability of the parameters (Park et al., 2021).

The BSR model also assumes that the decision-maker determines a target number of pumps before each trial that does not change while the decision-maker is pumping. This is a crucial distinction from the EWMV model because the assumption that the target number of pumps is determined prior to pumping means that the decision maker is not considering the potential loss if the balloon bursts.

The decision-maker’s subjective utility of pumping on balloon *k* on pump *l*—where utility is defined as a power function (Tversky & Kahneman, 1992)—is given in Equation 5:


5 \[
{U_{kl}} = {(1 - p_k^{burst})^l}{(lr)^\gamma }
\]

where *r* is the amount of reward per successful pump and *γ* is the decision-maker’s risk propensity. We then take the first derivative of Equation 5 with respect to *l* and set it to zero in order to optimize the equation and determine what the decision-maker considers their ‘optimal’ target number of pumps on each trial *k* (*v*_k_):


6 \[
{v_k} = \frac{{ - \gamma }}{{{\mathrm{ln}}\left({1 - p_k^{burst}} \right)}}\,\,with\,\gamma \ge 0
\]

Here, the optimal target number of pumps (*v*_k_ on trial *k* is determined by the subjective probability that the balloon will not burst ( \[
1 - p_k^{burst}
\] ) and the decision-maker’s propensity for risk-taking (*γ*). Values for *γ* are estimated from the data and higher values indicate greater risk propensity. We then use the target pumps on balloon *k* to calculate the probability that the decision-maker will pump the balloon on pump *l* for trial *k* ( \[
p_{kl}^{pump}
\] ) using Equation 7:


7 \[
p_{kl}^{pump} = \frac{1}{{1 + {e^{\tau \left({l - {v_k}} \right)}}}}\,\,with\tau \ge 0
\]

where *v*_k_ is the optimal number of pumps and τ is the behavioral consistency (i.e., inverse temperature). Higher τ values indicate more consistent behavior while lower values would suggest behavior that is more random or erratic.

In sum, the BSR model estimates four variables of interest: prior belief of success (*φ*), learning rate (*η*), risk propensity (*γ*), and behavioral consistency (*τ*).

#### 3-parameter model (3par)

The 3par model assumes sequential decision-making but does not assume learning. It is therefore not a cognitively plausible account of behavior (as we expect individuals to learn over the course of the task), but it is a mathematically simpler baseline model that other models must statistically outperform. It estimates three free parameters—prior belief of burst (*θ*), risk propensity (*γ*), and behavioral consistency (*τ*)—from the data using modified versions of Equations 6 and 7. Because it assumes the decision-maker does not learn, we must modify Equation 6: a fixed prior belief parameter (*θ*) replaces \[
p_k^{burst}
\] and *v* replaces *vk* since, without learning, the optimal number of pumps also remains constant. These changes yield Equation 8, from which *θ* and *γ* are estimated.


8 \[
v = \frac{{ - \gamma }}{{{\mathrm{ln}}\left({1 - \theta } \right)}}\,\,with\,\gamma \ge 0
\]

Similar modifications are then made to Equation 7 to account for the no learning assumption: *v* is replaced with *vk* and \[
p_{kl}^{pump}
\] is replaced by \[
p_l^{pump}
\] . This produces Equation 9, from which *τ* is estimated:


9 \[
p_l^{pump} = \frac{1}{{1 + {e^{\tau \left({l - v} \right)}}}}\,\,with\,\tau \ge 0
\]

#### 2-parameter model (2par)

The 2par model assumes sequential decision-making, but not learning. However, in the 2par model we do not attempt to estimate prior belief (*θ*). Rather, the decision-maker’s prior belief is fixed at 0.01 across all balloons. This model represents the simplest iteration of the four models; a baseline model that other more complex models must outperform. So, the 2par no learning model estimates just two parameters—risk propensity (*γ*) and behavioral consistency (*τ*)—from the data. The value of *θ* in Equation 9 is fixed at 0.01 to produce Equation 10:


10 \[
v = \frac{{ - \gamma }}{{{\mathrm{ln}}\left({1 - \left[ {0.01} \right]} \right)}}\,\,with\,\gamma \ge 0
\]

Here, a *γ* value is estimated from the data and used to calculate the optimal number of pumps (*v*). This value for *v* is then incorporated into Equation 9 to calculate values for *τ*.

### Model implementation

All models were run separately for BD+, BD–, and HC groups. While it is possible that running models separately in distinct groups may exaggerate group differences in parameters, leading to some false positives, modeling all groups at once is even more likely to underestimate group differences, causing a greater proportion of false negative findings (Valton, Wise & Robinson, 2020). We thus chose to model the groups separately.

Parameters were estimated using hierarchical Bayesian estimation using the hBayesDM v1.1.1, a Python package for modeling for common decision-making tasks (Ahn et al., 2017). This package performs Markov-chain Monte Carlo (MCMC) sampling in PyStan (Carpenter et al., 2017). The present analysis used PyStan v2.19.1.1 running on Python 3.6. For all models, the likelihood of the data given the parameters was calculated using the likelihood function specified by Wallsten et al. (2005). We used weak default priors to have minimal impact on the data. All models were run separately for BD+, BD–, and HC groups using 4 separate MCMC chains of 4000-samples (2000 burn-in) each. Because Stan uses Hamiltonian Monte Carlo (HMC), we tuned sampling parameters (adapt_delta, stepsize, max_treedepth) to ensure zero divergences (Betancourt, 2017). Additional information about the specifics of model implementation and links to all code used are provided in Supplement 2.1.

### Model evaluation

#### Diagnostics

For nearly all parameters/models/groups, trace plots were well-mixed, rhat values were < 1.1 (indicating convergence; Gelman & Rubin, 1992), and autocorrelation was ~0 by a lag of 15. Collectively, this indicated parameters converged to target distributions. The exception was the 3par model, which showed poor posterior sampling distributions for γ for BD+ and BD– groups. Raw diagnostic outputs for each model are available at: https://osf.io/zjmy8/?view_only=4bd534b2c3db4304be941f9414541440.

#### Model comparison

Model comparison was performed by calculating the leave-one-out (LOO) information criterion (Vehtari et al., 2017) for each group/model. This was done using the ‘loo’ function of the ArviZ Python package (Kumar et al., 2019). Results of the model comparison are provided in Table 2. For BD+ and BD–, EWMV performed marginally better than BSR. For HC, BSR performed marginally better than EWMV for HC, but the LOO difference was well within the LOO standard error. Given that EWMV and BSR unanimously outperformed 3par and 2par models in terms of LOO, we excluded the 3par and 2par models.

**Table 2:** Model comparison: Leave-one-out (LOO) information criterion.


**Group**	**Model**	**LOO**	**SE**	Δ**LOO**

BD+	EWMV	1774.00	82.68	0

	BSR	1799.72	87.02	25.72

	Par2	1935.07	76.89	161.07

	Par3	1938.79	75.99	164.79

BD–	EWMV	1497.36	70.89	0

	BSR	1512.64	73.54	15.28

	Par3	1612.30	77.64	114.94

	Par2	1614.21	78.27	116.85

HC	BSR	2854.09	114.28	0

	EWMV	2855.26	107.53	1.17

	Par2	3119.08	129.02	264.99

	Par3	3121.87	128.22	267.78


*Note*: Lower LOO values are indicative of better model performance. LOO = Leave-one-out Information Criterion; SE = LOO standard error; BD+ = bipolar disorder (BD) with prior substance use disorder (SUD); BD– = BD without prior SUD; HC = healthy comparisons; EWMV = Exponential-Weight Mean-Variance model; BSR = Bayesian Sequential Risk-Taking Model; Par2 = 2-parameter (no learning) model; Par3 = 3-parameter model (no-learning; estimates prior belief).

#### Posterior predictive checks

Posterior predictive checks were used to test the accuracy of predicted values produced by EWMV and BSR models for all groups. Predicted values were obtained using the ‘inc_postpred’ model specification from the hBayesDM package. We obtained 8000 MCMC samples of predicted behavior (i.e., 4000 samples [minus 2000 burn-in] by 4 MCMC chains) at all pump opportunities for all balloons. From this, we calculated the predicted adjusted average pumps (excluding samples where predictions exceeded actual breakpoints) and the 90% highest density interval (HDI) of the predicted distribution per subject. Then, we compared these values to the actual adjusted average pumps for each subject on the BART to assess the ability of each model to predict observed trial-level behavior.

Complete outputs for posterior predictive checks are available in Supplement 3.1. Results showed that observed values for EWMV and BSR models were strongly correlated with predicted values (all Pearson *r*’s *≥* 0.995), indicating both models had high predictive accuracy.

#### Model selection

The EWMV model outperformed the BSR model within BD+ and BD– groups in terms of LOO values; in the HC group, LOO differences between EWMV and BSR were negligible. EWMV also showed excellent predictive accuracy in all three groups according to posterior predictive checks. For these reasons, we selected the EWMV model as our winning model and use it to examine group differences. In the supplement, we examine group differences with the BSR. Largely the same conclusions are reached with the two models, but we report differences when they arise.

#### Parameter recovery

Park et al. (2021) showed good parameter recovery performance for the EWMV model using a 30-trial version of the BART, but the present study employed a shorter 20-trial version of the task. Therefore, we performed a simulation-based model recovery to evaluate how well the EWMV parameters could be recovered using data from only 20 trials.

Complete details and results of parameter recovery are presented in Supplement 3.2. In summary, results indicated that in general EWMV parameter values can be recovered from a 20-trial BART, but difficulties recovering precise values can arise when: 1) participants show highly deterministic behavior, and 2) posterior distributions of certain parameters are narrow. In those cases, we were still able to recover group differences observed in the present study (that we report in ‘Results’). Based on these findings, one should interpret the precise values of EWMV parameters cautiously (using only 20-trials), but can have confidence in the validity of the group differences it identifies. Because the goal of the present paper is to primarily evaluate group differences in model parameters, we conclude that using EWMV parameters derived from a 20-trial BART is a suitable means of achieving this.

### Statistical analyses

Statistical analyses were performed in RStudio (version 1.4.1717) (R Core Team, 2013). Group differences on model parameters were assessed for all parameters by calculating the 90% highest density interval (HDI) of the posterior differences between groups using the ‘HDIofMCMC’ function in the hBayesDM R package (Ahn et al., 2017). This gives a reliable interval of the posterior differences between two groups for any given parameter. But, we denote a 90% HDI of differences that does not contain zero as a credible difference in the posteriors of two groups for a given parameter.

For traditional BART measures, self-report questionnaires, and neuropsychological assessments, group differences were assessed via one-way ANOVAs. Individual (mean) estimates were extracted for each participant for all parameters and subjected to correlational analysis. Correlations were used to examine relationships between model parameters and measures of motivational processes (SSS, BIS/BAS) and executive functioning. Although the EWMV model had difficulty recovering *precise* parameter values for certain combinations of values, parameter recovery results suggest that this was due to a small bias across values where we would expect the order of values to be preserved. Thus, we used Spearman’s correlations (using functions from ‘ggpubr’ [Kassambara, 2020], ‘ggstatsplot’ [Patil, 2021]), which relies on ranks rather than precise values. Original participant data is not openly available because participants did not provide permissions for public upload, but analysis code is available at https://osf.io/zjmy8/?view_only=4bd534b2c3db4304be941f9414541440 to promote transparency and replicability. Analysis outputs are also provided in the supplement.

## Results

The groups were well-matched in terms of age, sex, and education. BD+ and BD– did not differ in terms of diagnosis distribution (i.e. BD I, II, NOS), use of psychotropic medication, or mood symptoms. Descriptive statistics of participant characteristics are summarized in Table 3.

**Table 3:** Characteristics of the sample.


	BD+ (*n =* 18)	BD– (*n =* 15)	HC (*n =* 33)	GROUP DIFFERENCES		
			
	M (SD)	M (SD)	M (SD)	*F/t/χ^2^*	*p*	*Post-hoc*

*Demographic*						

Age (years)	36.5 (10.9)	29.7 (9.5)	33.5 (10.3)	1.76	0.180	

Sex (% female)	38.9	66.7	57.6	2.79	0.248	

Education (years)	14.9 (2.6)	15.4 (4.2)^a^	16.0 (1.9)	0.96	0.390	

*Clinical*						

Psych Meds (%)	77.8	93.3		1.54	0.215	

Antidepressant (%)	27.8	46.7		1.26	0.261	

Antipsychotic (%)	44.4	26.7		1.12	0.291	

Benzodiazepine (%)	38.9	20.0		1.38	0.240	

Mood Stabilizer (%)	61.1	73.3		0.55	0.458	

Stimulant (%)	11.1	6.7		0.20	0.658	

Diagnosis				0.55	0.761	

BD I (%)	83.3	73.3				

BD II (%)	11.1	20.0				

BD NOS (%)	5.6	6.7				

YMRS	2.8 (2.1)	1.7 (1.9)		–1.48	0.149	

HAM-D	3.4 (2.9)	2.7 (2.5)		–0.76	0.455	

*Self-report*						

BIS	20.4 (3.3)	20.9 (4.0)	18.2 (3.8)	3.61	0.033*	HC < BD–

BAS-Reward	17.2 (2.2)	17.9 (2.2)	17.5 (1.6)	0.54	0.587	

BAS-Fun	11.1 (3.1)	11.1 (3.2)	11.0 (2.2)	0.02	0.984	

BAS-Drive	11.2 (3.3)	11.4 (2.5)	10.7 (2.8)	0.35	0.705	

SSS-Disinhibit	5.2 (2.9)	5.1 (2.3)	3.8 (2.6)	1.95	0.151	

SSS-Thrill	5.7 (3.5)	5.2 (2.9)	5.7 (2.8)	0.18	0.838	

SSS-Bored	3.8 (1.8)	2.8 (2.0)	2.0 (1.4)	6.82	0.002**	HC < BD+

SSS-Exper	6.3 (1.7)	5.6 (2.3)	5.5 (1.6)	1.18	0.313	

*Neuropsychological*						

Exec Func	–0.5 (1.1)^b^	0.2 (1.0)	0.2 (0.9)	2.97	0.059	


*Note*: BD+ = bipolar disorder (BD) with prior substance use disorder (SUD); BD– = BD without prior SUD; HC = healthy comparisons; M = mean; SD = standard deviation; Psych Meds = taking psychotropic medication; BD NOS = BD not otherwise specified; YMRS = Young Mania Rating Scale; HAM-D = Hamilton Depression Rating Scale; Exec Func = measure of executive function from Principal Components Analysis on Trail-Making Test (Part-B; TMT-B), Category Verbal Fluency (CVF), Stroop, Tower of London (ToL), and Digit Span Backward (DSB) scores; BIS = behavioral inhibition; BAS = Behavioral Activation Scale (reward sensitivity, fun-seeking, drive); SSS = Sensation Seeking Scale (disinhibition, thrill/adventure seeking, boredom susceptibility, experience seeking). ^a^ Based on 14 participants due to missing data. ^b^ Based on 17 participants due to missing data. * *p* < .05 ** *p* < .01.

### EWMV parameters

**Figure 2:**
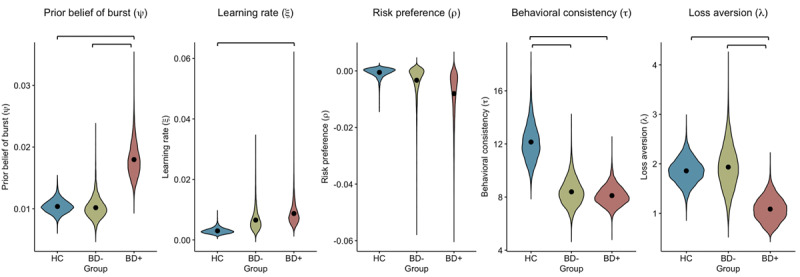
Group differences in EWMV parameters indexing the mechanisms of risk-taking. *Note*: Violin plots are based on 8000 post warm-up MCMC samples of the posterior distributions. Horizontal bars at top indicate credible differences between two groups based on 90% HDI of posterior differences for the given parameter. Dots indicate the mean of the posterior. BART = Balloon Analogue Risk Task; EWMV Model = Exponential-weight mean-variance model; *ψ* = prior belief of burst; *ξ* = learning rate; *ρ* = risk preference; *τ* = behavioral consistency; *λ* = loss aversion; BD+ = bipolar disorder with lifetime substance use disorder (SUD); BD– = bipolar disorder without lifetime SUD; HC = healthy comparisons.

In this section the 90% HDI of the posterior group differences (for each parameter and group pairing) is presented in brackets, followed by the mean of the posterior difference. Intervals that do not contain zero are interpreted as a ‘credible difference’ and the mean gives an indication of effect size. Credible posterior group differences were found for several EWMV parameters (Figure 2).

#### Prior beliefs

In terms of prior beliefs that the balloon would explode, BD+ exhibited more pessimistic prior beliefs (*ψ*) than BD– [0.003 0.01; *M* = 0.008] and HC [0.003 0.01; *M* = 0.008], while BD– and HC did not show credible differences [–0.003 0.003; *M* = –0.0002].

#### Learning rate

Unexpectedly, BD+ had credibly *higher* learning rates (*ξ*) than HC [0.0004 0.01; *M* = 0.006], though did not credibly differ from BD– [–0.005 0.01; *M* = 0.002]. BD– and HC did not credibly differ [–0.002 0.009; *M* = 0.004]. This may also indicate less certainty in prior beliefs among the BD+ group relative to HC.

#### Risk preference

Contrary to our hypothesis, none of the groups exhibited credible differences in estimates of risk preference *ρ*: BD+ vs. HC = [–0.02 0.003; *M* = –0.007; BD+ vs. BD– = [–0.02 0.01; *M* = –0.005]; BD– vs. HC = [–0.01 0.004; *M* = –0.003].

#### Behavioral consistency

As hypothesized, BD groups had credibly lower behavioral consistency *τ* estimates than HC (BD+ vs. HC = [–6.37 –1.32; *M* = –4.034]; BD– vs. HC = [–6.47 –0.94; *M* = –3.749]). This difference indicates that both BD+ and BD– made more inconsistent decisions than HC during risk-taking on the BART and replicates Yechiam et al.’s (2008) results. BD+ and BD– did not differ in terms of behavioral consistency (*τ* [–2.36 1.68; *M* = –0.286]).

#### Loss aversion

As hypothesized, BD+ had credibly lower loss aversion estimates (*λ*) than BD– [–1.67 –0.02; *M* = –0.849] and HC [–1.37 –0.23; *M* = –0.772]. BD– and HC did not show credible differences [–0.73 0.91; *M* = 0.076].

#### Correlations

On the BIS/BAS self-reports, reduced aversion to negative events on the BIS correlated with higher risk preference estimates (*ρ*) from the EWMV model, rho = –0.44, *p* < 0.01 (Supplement 4.2). BIS scores also varied between groups (*F* = 3.61, *p* < 0.05; post-hoc = BD– > HC). BAS self-report scores did not relate to EWMV model parameters (Supplement 4.2) and did not vary by group (Table 3). For the SSS, greater self-reported boredom susceptibility was associated with more pessimistic prior beliefs (*ψ*; rho = 0.25, *p* < 0.05), higher learning rates (*ξ*; rho = 0.32, *p* < 0.05), reduced behavioral consistency (*τ*; rho = –0.28, *p* < 0.05), and lower loss aversion (*λ*; rho = –0.35, *p* < 0.05)—consistent with the profile of the BD+ group. Indeed, SSS-boredom self-reports differed between groups (*F* = 3.61, *p* < 0.05), with post-hoc test indicating BD+ > HC. Remaining SSS subscales did not correlate with EWMV model parameters (Supplement 4.2) and did not vary by group (Table 3). Finally, poorer executive functioning on neuropsychological tests was related to lower behavioral consistency (*τ*; rho = 0.3, *p* < 0.05) and higher learning rates (*ξ*; rho = –0.28, *p* < 0.05), although executive functioning performance did not differ between groups (*F* = 2.97, *p* > 0.05).

#### Post-hoc comparison to BSR parameters

As a point of comparison for the results from the EWMV model, we re-ran analyses (post-hoc) using parameters from the BSR model. Results are provided in Supplement 6.1 to 6.3. To summarize, group differences and correlation results of the EWMV model were largely the same with those based on the BSR model. The main exceptions were: (1) loss aversion (*λ*) differences found with the EWMV model were instead captured by the risk propensity parameter (*γ*) of the BSR model, and (2) the BSR model was not able to distinguish between BD groups and HC based on behavioral consistency.

### Traditional BART measures

Groups did not differ significantly in terms of traditional BART measures (Supplement 5.1), which included adjusted pumps (*F* = 1.70, *p* > 0.05), total points (*F* = 1.04, *p* > 0.05), and total pops (*F* = 1.45, *p* > 0.05). Within the full sample, traditional BART measures also did not significantly correlate with BIS/BAS or SSS self-reports or neuropsychological tests of executive functioning (Supplement 4.2).

#### Simulations (post-hoc)

To better understand how differences in the EWMV parameters translated into pump behavior, we performed a set of post-hoc simulations with the EWMV model for each credible group difference we observed. For each simulation, for each subject, we defined a set of plausible values for model parameters by randomly sampling from normal distributions (with a mean and SD matching the distribution of a group posterior for that given parameter) truncated by the value constraints imposed on parameters. These values were used to generate trial-level pump behavior for a simulated agent using the EWMV model. The total number of agents generated for a given simulation equaled the sample size of the group in question. From this, we calculated the adjusted average number of pumps, averaged across all simulations. Using these procedures, we first used the EWMV model to perform 50 simulations of pump behavior based on true parameter values. Second, we used the EWMV model to perform 50 simulations of pump behavior based on parameter values that had been adjusted for the group difference in question. For instance, BD+ had credibly higher estimates for prior beliefs (*ψ*) than HC and the mean of the difference in their posterior distributions was 0.008. Therefore, we performed 1) 50 simulations for BD+ with prior belief (*ψ*) estimates *reduced* by 0.008, and 2) 50 simulations for HC with prior belief (*ψ*) estimates *increased* by 0.008. This gave us an indication of the level of behavioral change that a difference of the magnitude observed could produce.

Complete simulation results are presented in Table 4. Results indicated that the credible difference we observed in prior belief (*ψ*) parameters between BD+ and HC/BD– produced changes in behavior by ~3–15 pumps. Changing true values for learning rate (*ξ*) parameters by a magnitude of the difference between BD+ and HC altered the adjusted pumps of the group by ~5–6 pumps. Shifting behavioral consistency (*τ*) values (relative to true parameter values) by a magnitude of the difference observed between BD+/BD– and HC changed the adjusted pumps of the group by ~2–12 pumps. Finally, adjusting true values of loss aversion (*λ*) parameters by a magnitude of the differences observed between BD+ and HC/BD– produced some of the most pronounced changes in behavior, changing the group adjusted pumps by ~8–21 pumps.

**Table 4:** Post-hoc simulation results.


	OBSERVED DIFFERENCES		SIMULATIONS		

	*DIRECTION*	*MEAN DIFFERENCE*	*GROUP*	*ADJUSTMENT (ΔPARAMETER VALUE)*	*OUTCOME (ΔADJ. PUMPS)*

Prior Belief (*ψ*)	BD+ > BD–	0.008	BD–	Increased *ψ* by 0.008	Decreased **3 pumps**

			BD+	Decreased *ψ* by 0.008	Increased **4 pumps**

	BD+ > HC	0.008	HC	Increased *ψ* by 0.008	Decreased by **15 pumps**

			BD+	Decreased *ψ* by 0.008	Increased by **4 pumps**

Learning Rate (*ξ*)	BD+ > HC	0.006	HC	Increased *ξ* by 0.006	Decreased by **6 pumps**

			BD+	Decreased *ξ* by 0.006	Decreased by **5 pumps**

Behavioral Consistency (*τ*)	BD+ < HC	–4.034	BD+	Increased *τ* by 4.034	Increased by **8 pumps**

			HC	Decreased *τ* by 4.034	Decreased by **11 pumps**

	BD– < HC	–3.749	BD–	Increased *τ* by 3.749	Increased by **2 pumps**

			HC	Decreased *τ* by 3.749	Decreased by **12 pumps**

Loss Aversion (*λ*)	BD+ < BD–	–0.849	BD–	Decreased *λ* by 0.849	Increased by **21 pumps**

			BD+	Increased *λ* by 0.849	Decreased by **11 pumps**

	BD+ < HC	–0.772	HC	Decreased *λ* by 0.772	Increased by **17 pumps**

			BD+	Increased *λ* by 0.772	Decreased by **8 pumps**


*Note*: Mean difference = to calculate the ‘mean difference’ we took the difference between the mean posterior distributions for both groups (for a given parameter) and determined the mean of the resulting distribution of differences; Adjustment (Δ Parameter Value) = indicates the direction and magnitude the parameter was changed for simulations; Outcome (Δ Adj. Pumps) = change in the overall adjusted average number of pumps that resulted from the change in parameter value shown in the ‘adjustment’ column (relative to adjusted pumps using true parameter values), averaged across 50 simulations.

In general, increasing/decreasing parameter values produced changes in pump behavior in expected directions. For instance, increasing prior belief (*ψ*) values (i.e., more ‘pessimistic’ expectations), learning rates (*ξ*), and loss aversion (*λ*) all led to decreases in pump behavior. However, contrary to expectation, increasing behavioral consistency (*τ*) led to apparent increases in pump behavior. This result has been found previously and represents an artifact of the adjusted BART score that modeling approaches can help to address (see Pleskac, 2008 for discussion).

## Discussion

The present analysis parsed the cognitive processes underlying risk-taking behavior on the BART using the Exponential-Weight Mean-Variance (EWMV) model. We uncovered several mechanistic distinctions in risk-taking in euthymic BD: some were BD–specific and others were unique to BD with prior SUD; some may confer vulnerabilities for risk-taking in BD and others may offset them. These results highlight the value of computational modeling in mechanistic studies of risk-taking in complex disorders like BD.

In partial alignment with hypotheses based on existing findings (Martinez-Aran et al., 2004; Mur et al., 2007; Ryan et al., 2012), we found that a reduction in the consistency of choice behavior (τ) during risk-taking was general to BD regardless of SUD history. While these differences were not also exacerbated in BD+ as originally anticipated, behavioral consistency (and learning rate parameters) were related to performance on neuropsychological tests of executive functioning, offering preliminary evidence suggesting that these parameters capture meaningful facets of cognitive control during risk-taking. Taken together, these results suggest that reduced behavioral consistency is a feature of BD (irrespective of SUD history) that may be related to general executive control deficits observed in the disorder. Future studies using larger samples and a greater number of trials are needed to replicate and confirm this notion.

Our finding of reduced behavioral consistency among the BD groups aligns with Yechiam et al.’s (2008) finding of reduced behavioral consistency in manic-BD during the Iowa Gambling Task, relative to HC. However, they found no reductions in behavioral consistency in euthymic-BD as we did. This discrepancy may be due to the use of different tasks and perhaps also the modeling approach. Bayesian hierarchical modeling, the approach used in our study, estimates parameters more accurately than non-hierarchical ones (Rouder et al., 2005; e.g., maximum likelihood estimation used by Yechiam et al.) because pooling data over groups reduces variability of parameters. In our case, this may have enabled detection of subtle group differences. Intriguingly, Park et al. (2021) found very little difference in behavioral consistency between HC and SUD samples (who presumably were non-BD). These findings together suggest that reductions in behavioral consistency may be specific to BD, not SUD. Given emerging evidence of the important role played by behavioral consistency in predisposing altered risk-taking in BD (regardless of substance use history), longitudinal investigations are needed to elucidate how executive functioning and behavioral consistency interact with mood states over time in BD and contribute to risk-taking behavior.

We also examined underlying motivational processes relevant to risk-taking behavior. The ‘motivational parameters’ of the EWMV model (i.e., risk preference and loss aversion) were significantly associated with self-reported aversion to negative events and sensation seeking, providing support to the relevance of these parameters to motivational processes underlying risk-taking. While we did not find increased risk preferences in either of the BD groups, BD+ exhibited reduced loss aversion relative to both BD– and HC, and this reduction was not present in BD–. This suggests that SUD history in BD may reflect a trait-level vulnerability characterized by altered reward processing, which in turn predisposes risk-taking behavior (see also Stout et al., 2004; Park et al., 2021). Fittingly, Holmes et al. (2009) found reductions in risk-taking on the BART (i.e., fewer pumps) after a burst in HC and BD– but not BD+, suggesting a persistently high drive for reward in BD+ that was not deterred by negative feedback. Considered together, these findings suggest that a history of SUD among those with BD may reflect certain vulnerabilities for risk-taking (e.g., due to reduced loss aversion) and thus higher risk for poorer outcome. However, these results should be interpreted cautiously because we did not include a control group of individuals with lifetime SUD but not BD. In addition to examining added control groups, future work should also employ tasks with a larger number of trials, based upon which loss aversion parameters can most reliably recovered.

If BD groups possess trait vulnerabilities for risk-taking (particularly BD+), we should address why our groups showed similar overall levels of risk-taking on the BART in terms of adjusted number of pumps. One reason is that opposing factors were also observed in both BD groups that may have offset these vulnerabilities. For instance, our results indicated that BD+ appeared more cautious at the beginning of the task as indicated by more pessimistic prior beliefs but simultaneously had lower behavioral consistency and loss aversion. Together this combination of alterations can cancel out each other and result in little observed differences at the aggregate behavioral level. However, this combination of alterations would imply specific behavioral changes over the course of the task. Specifically, the more pessimistic prior beliefs in the BD+ group would suggest that they would pump less in the early trials of the BART and over time increase their level of risk taking due to their lower levels of loss aversion and faster rate of learning. This pattern was exactly what we observed in our data. We examined the adjusted pumps in the first, second, and last thirds of the trials in our sample, and found that BD+ were cautious and pumping on average about 38 times (compared to 45 in HC) in the first third trials of the task; by the final third, they pumped on average 44 times (compared to 44 in HC) (see Supplement 7.1 to 7.3). Although these group differences did not reach statistical significance at the observed behavioral level, the latent processes isolated by the computational model parameters showed credible group differences. Future study should examine the interplay between these motivational vulnerabilities and (possible) protective factors in their contribution to observed risk-taking in BD.

One surprising finding was that BD+ exhibited the highest learning rates on the BART, followed by BD– and HC. While this result should be interpreted cautiously, it raises an interesting question: are higher learning rates always favorable? Initially, we expected that higher learning rates would be associated with improved executive functioning performance on neuropsychological tests and the highest learning rates would occur in HC. But, in our case BD+ exhibited the higher learning rate and those were associated with poorer executive functioning. It is useful, however, to revisit what this ‘learning rate’ parameter actually indexes. It refers to the rate at which prior beliefs are updated based on observations over the course of the task. At least in the BART, as we discussed above, BD+ showed the greatest degree of change in their behavior across the balloons. In comparison, the low learning rates from HC and BD– simply indicate that these groups did not update their beliefs very much and as such, their adjusted pumps stayed relatively stable over blocks (as seen in Supplement 7.3). One possible interpretation is that in the absence of altered motivational processes, BD– and HC were able to maintain their prior beliefs throughout the task, even after the excitement of banking points on initial trials. Perhaps, BD+ were unable to maintain more conservative initial beliefs due to altered reward processing, which led them to more readily update their mindset and take more risks over time. A second possible interpretation considers that learning rates also reflect the certainty of one’s prior beliefs. Decision-makers with less confidence in their prior beliefs will also update their expectations more quickly than those with greater confidence in their expectations. It is therefore possible that while BD+ showed more pessimistic prior beliefs, that they held less confidence in these expectations, leading them to update their beliefs more quickly as they gained experience. However, these are merely speculative. Future studies using sequential risk-taking paradigms (like the BART) are needed to shed light on how risk-taking behavior in BD changes over time as a function of alterations in the underlying processes.

It is noteworthy that computational parameters (from both the EWMV and BSR models), but not traditional measures (adjusted pumps, pops, points), revealed group differences (distinguishing between BD and HC, as well as BD+ and BD–) and relationships with self-reports and neuropsychological tests of executive functioning. It is possible that the theoretical mechanisms giving rise to behavior can be decomposed and extracted only by examining trial-level data, rather than averaging behavior across the entire task. This is consistent with previous accounts of increased sensitivity of computational approaches to subtle nuances in behavior than mean-based metrics (Huys et al., 2016; Yechiam et al., 2008), underscoring the value of using mathematical models to understand behavior in psychopathology research.

The results of this study should be interpreted in light of several limitations. First, the sample size of the present study was modest and future studies with larger samples are needed to not only replicate the findings, but also provide greater statistical power to examine effects or relationships not examined in this study (e.g., sex differences, relationship with illness chronicity). Second, while focusing on the euthymic phase of BD had the advantage of revealing vulnerabilities not confounded by mood states, we were not able to address the questions whether and how risk-taking changes during acute mood episodes, and whether the model parameters (or their fluctuations over time) are predictive of future mood episodes like changes in BIS/BAS self-reports are (Alloy et al., 2008). Longitudinal studies could expand on these findings and render clinically relevant knowledge to guide treatment planning. Third, data has shown that behavior in the BART is relatively stable and reliable (Lejuez et al., 2007; White et al., 2008). However, some recent work has questioned this, indicating that standard behavioral risk-taking measures show poor reliability over time (Frey et al., 2017). One possible explanation for theses reliability differences is the probabilistic nature of the BART, which makes it a risk-taking task but also can lead to very different experiences between participants and thus insert additional noise into the measurement (Schürmann et al., 2019). We worked to minimize these differences by fixing the burst points between participants and modeling participants at the individual level using the trial level data via the cognitive model, while also arranging a set of practice trials to allow for greater stability of performance during the task itself. Nevertheless, future work should establish the reliability of key parameters using the BART and other risk-taking tasks. Fourth, we did not include a control group with lifetime SUD but not BD. Inclusion of such a group in future investigations would help examine which aspects of our findings were unique to SUD in the context of BD (i.e., BD+) and which were characteristic of prior SUD more generally. Fifth, the current study was not preregistered because we chose this specific computational analysis approach after the data was collected. Sixth, there were differences in LOO values between BD groups and HC. This may indicate small differences in strategy between BD and HC (i.e., behavioral inconsistency within BD groups and/or greater exploration in BD+). Additional studies should explore the possibility of disparate decision-making strategies to further clarify this. Seventh, we recognize the possibility that findings related to decision consistency may be a function of increasing general psychiatric vulnerability (and not specific to BD and SUD, per se; [e.g., Moutoussis et al., 2021]) and future work should compare across diverse diagnostic groups to examine this directly. Eighth, our simulation results indicated that we could confidently interpret differences identified by the EWMV model for a 20-trial BART, which allowed us to accomplish our goals of assessing group differences on model parameters. However, we recognize that this is not the goal of all studies and, consequently, using the EWMV model on a 20-trial BART may not always be appropriate. Therefore, future work (especially those interested in loss aversion and interpreting precise values of parameters) should employ tasks with a larger number of trials. Ninth, participants were trained with a variable distribution of burst points (during the initial practice), while the models used assumed a flat probability of the balloon bursting on each trial. One could argue that the probability of the balloon bursting should match this training phase (i.e., modeling a variable burst probability per trial rather than a constant one), but the use of a flat probability is well-supported by prior evidence (see Supplement 7.4 for complete discussion). Tenth, as we built on past work showing that the EMWV showed better prediction of data out of sample (Park et al., 2021) and our study was not designed to carry out extensive model comparisons, we did not perform a formal model recovery analysis by simulating data using the EMWV and BSR models within our study design and checking that our model comparison approach can infer the correct model in the case of each group. Taken together, pre-registered replications with larger sample sizes, more trials, additional populations, and more observations would help further validate and clarify the findings reported here.

In summary, the present study used a computational (EWMV) model to characterize the cognitive processes underlying behavior in risky decision-making in euthymic BD. The EWMV model could distinguish between groups based on both BD diagnosis and history of SUD, showing that more inconsistent behavior during risk-taking was general to BD, but additional differences (reduced loss aversion, more pessimistic prior beliefs, and a potentially maladaptive tendency to update beliefs based on recent experience) were unique to BD with history of SUD. Taken together, our findings suggest that reduced behavioral consistency is a crucial feature of risky decision-making in BD and that SUD in BD may reflect additional trait vulnerabilities (e.g., reduced loss aversion) contributing to risky behavior even when mood symptoms and substance use are in remission.

## Additonal File

The additonal file for this article can be found as follows:

10.5334/cpsy.61.s1 Supplemental Information. Supplementary materials, methods, and results.
