# Supplementary material for: Mathematical Modeling of Risk-Taking in Bipolar Disorder: Evidence of Reduced Behavioral Consistency, With Altered Loss Aversion Specific to Those With History of Substance Use Disorder
Source: Comput Psychiatr. 2022 May 24;6(1):96–116. doi: 10.5334/cpsy.61 (PMC9897236; doi:10.5334/cpsy.61)
Supplement: Supplemental Information. — Supplementary materials, methods, and results. [file cpsy-6-1-61-s1.pdf]

Lasagna et al. (2022) Supplemental Information

Code available at: https://osf.io/zjmy8/?view_only=4bd534b2c3db4304be941f9414541440

1. Descriptions of self-report and neuropsychological measures

1.1. Behavioral Inhibition/Activation System Scale. The Behavioral Inhibition and Activation System (BIS/BAS) scale is a 20-item self-report measure which captures trait responsivity to rewarding and threatening experience (Carver & White, 1994). Statements are rated on a 4-point scale from "Strongly Agree" to "Strongly Disagree" and assess activity of two neuropsychological systems thought to motivate behavior: (1) Behavioral Inhibition System (BIS), which inhibits behavior to evade negative consequences, and (2) Behavioral Activation System (BAS), which propels responsivity to rewarding aspects of positive experience and drives goal-directed pursuit (Gray, 1981; Pickering et al., 1997). Items gauge BIS activity (e.g., "I feel worried when I think I have done something poorly") and three domains of the BAS: reward response (e.g., "When I get something I want, I feel excited and energized"), fun-seeking (e.g. "I'm always willing to try something new if I think it will be fun"), and drive (e.g., "When I want something I usually go all-out to get it").

1.2. Sensation Seeking Scale. The Sensation Seeking Scale (SSS; Zuckerman et al., 1964) is a 40-item self-report scale developed to assess sensation seeking traits in 4 domains using a true-false response format: Disinhibition (e.g., "I like 'wild' uninhibited parties"), Boredom Susceptibility (e.g., "I have no patience with dull or boring persons"), Thrill/Adventure Seeking (e.g., "I often wish I could be a mountain climber"); Experience Seeking (e.g., "I like to explore a strange city or section of town by myself, even if it means getting lost").

1.3. Neuropsychological tests. Participants completed a battery of executive functioning tasks evaluating working memory, cognitive flexibility, inhibition, verbal fluency, and planning/problem-solving: Wechsler Adult Intelligence Scale – 4th Edition Digit Span Backward (DSB; Wechsler, 2008), Trail-Making Test Part B (TMT-B; Reitan, 1958), Stroop Color-Word Test (Stroop; Stroop, 1935), Categorical Verbal Fluency-Animal Naming (CVF; Rosen, 1980), and Tower of London (ToL; Anderson et al., 1996). Higher scores were indicative of greater executive function, with the exception of TMT-B where a higher score indicated poorer performance. A Principal Components Analysis (PCA) was performed on the z-scores of these measures in IBM SPSS Statistics Version 24 to derive a single executive functioning factor to be used in analyses. The sign of the z-score for TMT-B was reversed beforehand to ensure consistent directionality of all measures.

2. Notes on model code

2.1. Model code is available on OSF. Files (i.e., Stan file and Python code) for the BSR model were taken directly from the hBayesDM v1.1.1 Python package ('bart_par4'; Ahn et al., 2017). Model files for the 3par model were written based on specifications from Park et al. (2021). Those for the 2par model were based on custom model code implemented in hBayesDM. Model files for the EWMV model used here were adapted from the 'bart_ewmv' files in hBayesDM v1.1.1. We adapted them for several reasons. First, we modified the calculation of p_k^{burst} in the 'bart_ewmv' Stan file from hBayesDM v1.1.1. to match the equations presented in Park et al. (2021). Second, during initial test implementations of the EWMV model, we noted difficulties in sampling the posterior distribution for risk preference (ρ) in the BD+ group where estimates for loss aversion (λ) were exceptionally low (i.e., < ~0.25). Estimation of ρ becomes very tightly constrained and suffers with extremely low estimates for λ. To compensate, we constrained values for λ parameters to λ > 0.5 (originally λ > 0) and this resolved the sampling issue.

3. Model evaluation

3.1. Posterior predictive checks (EWMV and BSR)

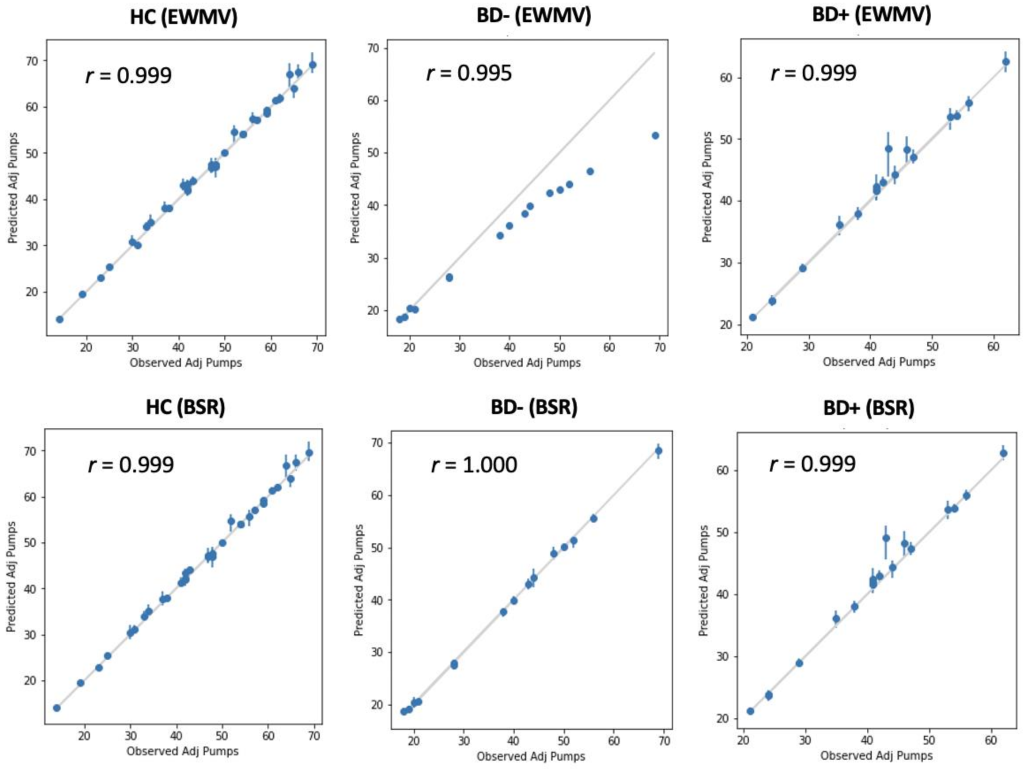

Note. Posterior predictive checks for EWMV (top) and BSR models (bottom). Error bars show 90% HDI of posterior predicted samples for each subject. BD+ = bipolar disorder (BD) with prior substance use disorder (SUD); BD- = BD without prior SUD; HC = healthy comparisons; EWMV = Exponential-weight mean-variance model (Park et al., 2021); BSR = Bayesian Sequential Risk-Taking Model (Park et al., 2021; Wallsten et al., 2005).

3.2. Parameter recovery (EWMV only). To conduct our parameter recovery, we used the posterior estimates of each of the groups' (BD+, BD-, HC) to generate new samples of data for each group. To do so, we took the group-level means and standard deviations and generated datasets for each group that had the number of (simulated) participants with each participant completing 20 trials of the BART that was used in this study (i.e., same explosion sequence). Then for each simulated dataset for each group, we fit the EMWV to the data using the same fitting process used in the main analysis. Due to computational and time constraints we deviated from the main analysis and used 1000 samples, 500 burn-in, 4 chains. We repeated this process 50 times. <u>*Recovering precise values*</u>. We found the percentage of simulations where the 95% HDI of simulated posteriors contained the true posterior mean (i.e., generating value for a given parameter). Results showed that ξ, ρ, τ, λ were recovered well for BD+ (86-98% recovery; see 3.2.1 and 3.2.2), but ψ was consistently underestimated (45% recovery). For BD- and HC, the opposite pattern was found: ψ was well-recovered (98% recovery for both), but the remaining parameters (ξ, ρ, τ, λ) were less reliably recovered (22-68% recovery). On its own, this seems to suggest that it is possible to recover all of the EWMV parameters based on only 20-trials of BART data, but that difficulties arise with the recovery of certain precise values. We determined that poor recovery of ρ, τ, and λ for BD- and HC is likely the result of two factors. For one, several HC participants exhibited highly deterministic behavior. Given that τ follows an exponential distribution with no upper boundary, this led to sampling in the extremes in the upper tails of the true posterior that was not captured in simulations (hence, the apparent 'underestimation' of τ for HC during parameter recovery). A second factor was that both BD- and HC had extremely narrow distributions for values of ρ, with HC being the more extreme of the two. Since estimation of τ and λ is also impacted by estimation of ρ, lack of variability in the true ρ posterior likely hindered the successful recovery of these

three parameters collectively. We noted that BD+, on the other hand, had more variability in the true ρ posterior, and these three parameters were recovered successfully. Together, this suggests that it is possible to recover the parameters of the EWMV model with only 20-trials, but we should interpret precise values of parameters cautiously. ***Recovering group differences.*** Given our study was focused on testing for differences between groups, we next addressed the question of how well we could distinguish between groups. Thus, across the 50 simulated datasets for each parameter for each group, we calculated the percentage of times that we recovered a credible difference. As shown below (see 3.2.3), we were able to reliably recover the differences we observed on actual data through simulations for ψ, ξ, and τ parameters. Group differences for λ were less reliably recovered, but this is likely because precise values for λ were accurately recovered for BD+ and λ recovered values were systematically underestimated for both BD- and HC (see 3.2.1). In cases where the original difference was *not* recovered according to our criteria, we were still able to verify that the direction of the difference was preserved (i.e., that it had not been reversed entirely). For example, we expected lower λ in BD+ relative to HC based on our results in the main text. So, for all simulations where this difference was *not* recovered, we verified that the group λ posterior for BD+ was indeed lower than HC (albeit to a lesser magnitude).This gave us additional evidence that less reliable difference recovery for λ was a matter of mere magnitude and not changes to the nature of the group difference. Together, this evidence gave us confidence in the validity of the differences identified by the EWMV model. ***Conclusions.*** In summary, results indicated that in general EWMV parameter values can be recovered from a 20-trial BART, but difficulties recovering precise values can arise when: 1) participants show highly deterministic behavior, and 2) posterior distributions of certain parameters are narrow. In those cases, we still recovered group differences observed in the present study. Based on these findings, we interpret the precise values of EWMV parameters cautiously, but are also confident in the validity of the group differences it identifies.

3.2.1. Results of simulation recovery of true parameter values for the EWMV model

Group	*Prior Belief (ψ)*	*Learning Rate (ξ)*	*Risk Preference (ρ)*	*Behavioral Consistency (τ)*	*Loss Aversion (λ)*
**BD+**					
True μ	.018	.009	-0.008	8.11	1.09
True 95% HDI	[.013 .024]	[.003 .016]	[-0.026 0.002]	[6.78 9.55]	[0.62 1.53]
Simulated μ	.022	.010	-.014	7.65	1.00
Simulated 95% HDI	[.018 .025]	[.007 .013]	[-0.029 -0.003]	[6.60 8.77]	[0.73 1.29]
% Recovered	45%	98%	94%	86%	98%
**BD-**					
True μ	.010	.007	-0.003	8.40	1.93
True 95% HDI	[.007 .014]	[.002 .013]	[-0.014 0.002]	[6.51 10.42]	[1.08 2.79]
Simulated μ	.011	.012	-.026	7.29	1.23
Simulated 95% HDI	[.009 .014]	[.006 .021]	[-0.057 -0.004]	[6.04 8.63]	[0.81 1.71]
% Recovered	98%	68%	60%	62%	22%
**HC**					
True μ	.010	.003	-0.001	12.15	1.86
True 95% HDI	[.008 .012]	[.001 .005]	[-0.004 0.002]	[9.54 14.91]	[1.34 2.35]
Simulated μ	.011	.004	-.008	10.11	1.38
Simulated 95% HDI	[.009 .012]	[.003 .006]	[-.017 .000]	[8.36 11.94]	[0.98 1.79]
% Recovered	98%	58%	64%	38%	40%

Note. Parameter recovery for Exponential-Weight Mean-Variance (EWMV) model based on 50 simulations. True μ = true posterior mean observed in real participant data (generating value for simulated data); True 95% HDI = highest density interval of the mean posterior observed in real data; % recovered = percentage of fits based on simulated datasets that recovered the true μ value (i.e., the 95% HDI of the simulated posterior contained the true μ value); simulated 95% HDI = upper and lower limits of 95% HDI for posterior averaged across all simulations; BD+ = bipolar disorder (BD) with prior substance use disorder (SUD); BD- = BD without prior SUD; HC = healthy comparisons.

3.2.2. Plots of simulation recovery of true parameter values for the EWMV model

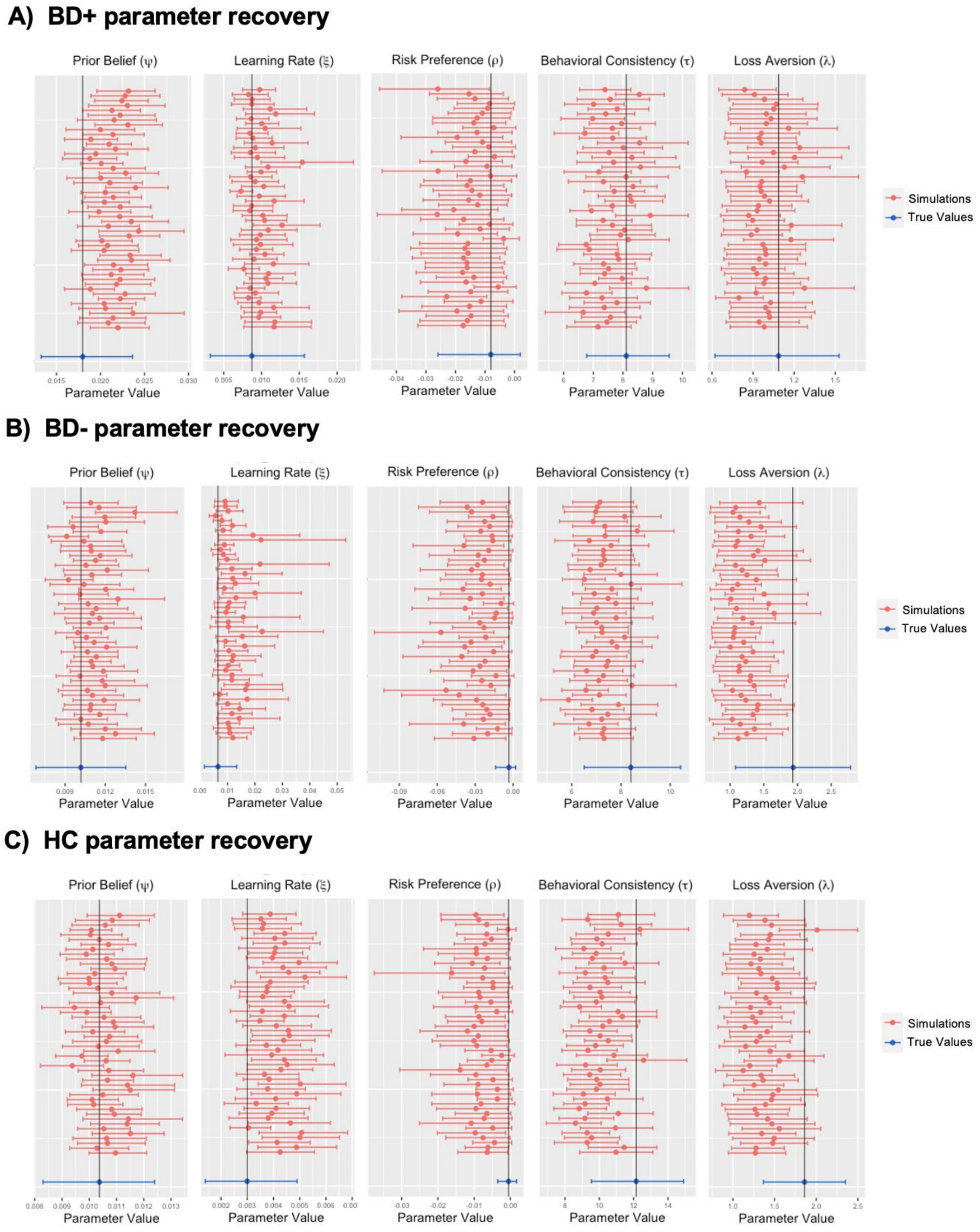

Note. Parameter recovery of the Exponential-Weight Mean-Variance (EWMV) model for BD+ (top), BD- (middle), and HC (bottom) based on 50 simulations. Dots indicate the mean of the posterior distribution. Errors bars depict the 95% highest density interval (HDI) of the posterior. The blue datapoint shows the true mean and 95% HDI of the mean posterior based on real data. The 50 datapoints above (in red) give the mean and 95% HDI of the posterior of 50 individual simulations. A given simulation recovers the generating value if the blue dot indicating the true posterior mean falls within the 95% HDI for the simulation (left/right error bars). A vertical line has been superimposed to facilitate interpretation; BD+ = bipolar disorder (BD) with prior substance use disorder (SUD); BD- = BD without prior SUD; HC = healthy comparisons.

3.2.3. Results of simulation recovery of observed group differences using the EWMV model

Comparison	*Prior Belief (ψ)*	*Learning Rate (ξ)*	*Risk Preference (ρ)*	*Behavioral Consistency (τ)*	*Loss Aversion (λ)*
BD+ vs. HC	100%	100%	6%	72%	36%
BD- vs. HC	6%	90%	18%	84%	4%
BD+ vs. BD-	100%	4%	4%	16%	10%

Note. Values show the percentage of model fits based on 50 simulated datasets that recovered group differences observed using the Exponential-Weight Mean-Variance (EWMV) parameters on real participant data. Shaded cells indicate where a credible difference was originally observed in the primary analyses. 'Credible differences' were based on 90% HDI of posterior differences for each parameter; intervals that did not contain zero were interpreted as a credible difference. BD+ = bipolar disorder (BD) with prior substance use disorder (SUD); BD- = BD without prior SUD; HC = healthy comparisons.

4. **Spearman's correlations.** We performed correlations between EWMV parameters, BSR parameters, and traditional BART measures to examine relationships among these variables. Although the EWMV model had difficulty recovering *precise* parameter values for certain combinations of values, parameter recovery results suggest that this was due to a general bias across values where we would expect the order of values to be preserved. Because of this, we used Spearman's correlations, which relies on ranks rather than precise values.

4.1. Spearman's correlations among EWMV parameters, BSR parameters, traditional BART measures

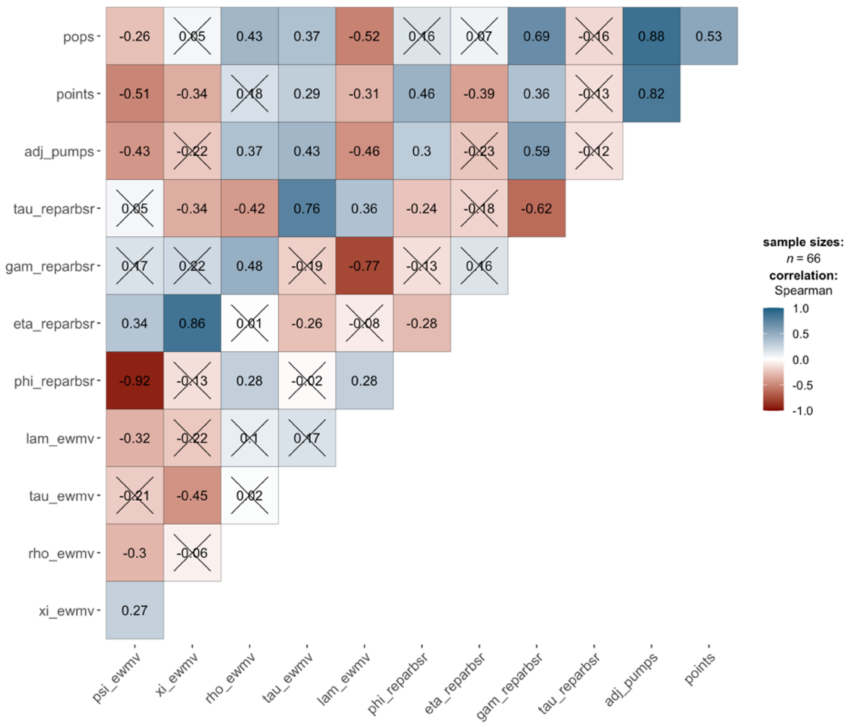

Note. Spearman's rho coefficients for correlations performed on full sample. Boxes containing an 'X' are not significant at $p < 0.05$ level (unadjusted); all others are significant at $p < 0.05$. Blue shading shows a positive correlation and red indicates a negative correlation. Correlations were exploratory and provided for illustrative purposes and they did not undergo correction for multiple comparisons. EWMV Model = Exponential-weight mean-variance model; BSR = Bayesian Sequential Risk-Taking Model; psi_ewmv = prior belief of burst (ψ); xi_ewmv = learning rate (ξ); rho_ewmv = risk preference (ρ); tau_ewmv = behavioral consistency (τ); lam_ewmv = loss aversion (λ); phi_bsr = prior belief of success (ϕ); eta_bsr = learning rate (η); gam_bsr = risk propensity (γ); tau_bsr = behavioral consistency (τ).

4.2. Spearman’s correlations between model parameters/traditional BART measures and self-report/neuropsychological data

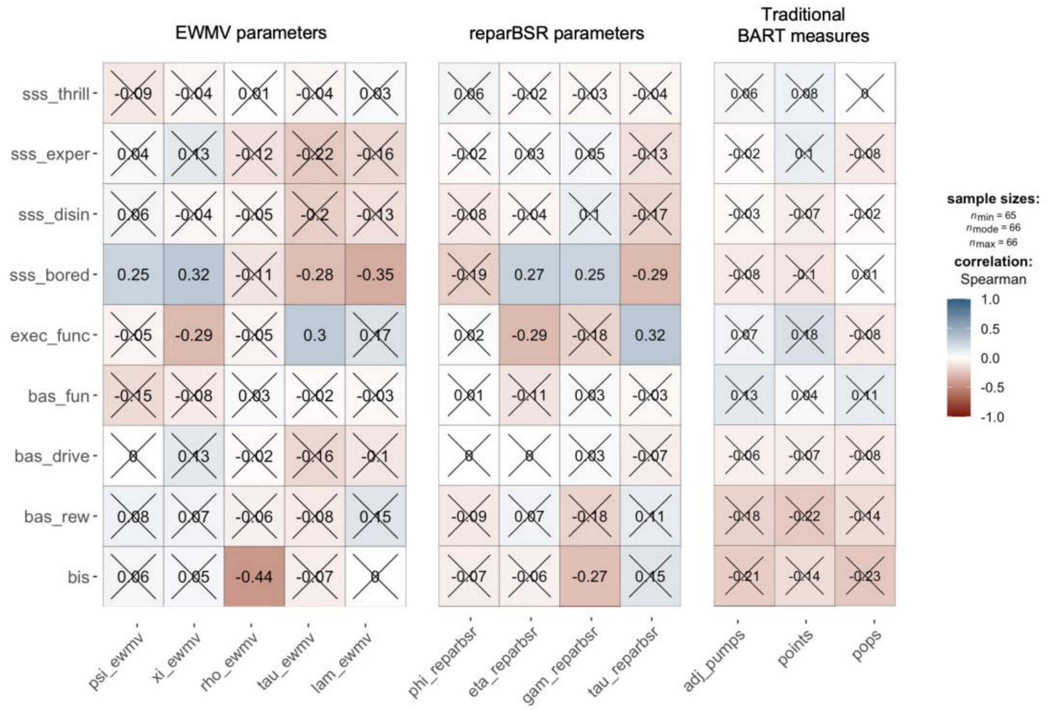

Note. Spearman’s rho coefficients for correlations performed on full sample. ‘X’ = not significant at $p <$ 0.05 (all others are significant). Correlations were exploratory/illustrative and, thus, did not undergo correction for multiple comparisons. EWMV Model = Exponential-weight mean-variance model; BSR = Bayesian Sequential Risk-Taking Model; psi_ewmv = prior belief of burst (ψ); xi_ewmv = learning rate (ξ); rho_ewmv = risk preference (ρ); tau_ewmv = behavioral consistency (τ); lam_ewmv = loss aversion (λ); phi_bsr = prior belief of success (ϕ); eta_bsr = learning rate (η); gam_bsr = risk propensity (γ); tau_bsr = behavioral consistency (τ); exec func = executive functioning; BIS = behavioral inhibition; BAS = Behavioral Activation Scale (reward sensitivity, fun-seeking, drive); SSS = Sensation Seeking Scale (disinhibition, thrill/adventure seeking, boredom susceptibility, experience seeking).

5. Traditional BART measures

5.1. Group differences on traditional BART measures

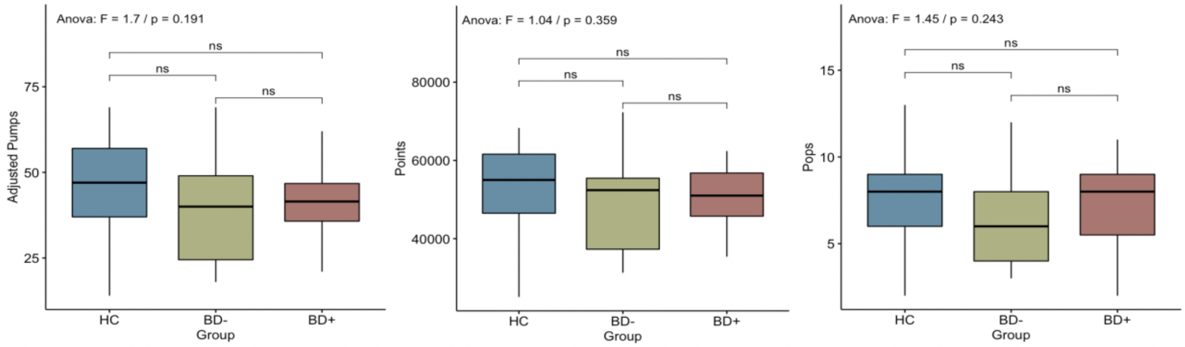

Note. ANOVA and post-hoc t-tests for traditional BART measures. BD+ = bipolar disorder (BD) with prior substance use disorder (SUD); BD- = BD without prior SUD; HC = healthy comparisons; ns = not significant at $p < 0.05$.

6. Analysis of BSR parameters

6.1. Background: Comparable parameters between EWMV and BSR models are: $\psi^{EWMV} \approx \phi^{BSR}$ [inverse relation]; $\xi^{EWMV} \approx \eta^{BSR}$; $\rho^{EWMV} \approx \gamma^{BSR}$; $\tau^{EWMV} \approx \tau^{BSR}$. Correlations among these are shown in Supplement 4.1.

6.2. Group differences – BSR parameters. Like the results from the EWMV analysis, the post-hoc BSR analysis showed that BD+ had more pessimistic prior beliefs (ϕ) than BD- and HC and that BD+ had lower behavioral consistency (τ) than HC. Unlike the EWMV model, BSR did not reveal any credible group differences for the learning rate parameter (η). Moreover, for BSR risk propensity (γ) we found credibly higher estimates in BD+ relative to BD- and HC.

90% highest density interval (HDI) of posterior differences per parameter

	BD+ minus HC	*BD+ minus BD-*	*BD- minus HC*
Prior belief of success (ϕ)	[-0.005 -0.0004; *M* = -0.003]	[-0.006 -0.001; *M* = -0.004]	[-0.001 0.003; *M* = 0.001]
Learning rate (η)	[-0.001 0.004; *M* = 0.001]	[-0.002 0.003; *M* = 0.001]	[-0.001 0.003; *M* = 0.001]
Risk propensity (γ)	[0.015 0.292; *M* = 0.152]	[0.074 0.363; *M* = 0.225]	[-0.185 0.044; *M* = -0.073]
Behavioral consistency (τ)	[-0.133 -0.010; *M* = -0.073]	[-0.070 0.038; *M* = -0.017]	[-0.120 0.009; *M* = -0.056]

Note. The 90% HDI of the posterior differences (for each parameter and group pairing) is presented in brackets, followed by the mean of the posterior difference. Intervals that do not contain zero are interpreted as a 'credible difference' (cells shaded in grey) and the mean gives some indication of the effect size. BSR = Bayesian Sequential Risk-Taking Model; BD+ = bipolar disorder (BD) with prior substance use disorder (SUD); BD- = BD without prior SUD; HC = healthy comparisons.

Group difference plots – BSR parameters

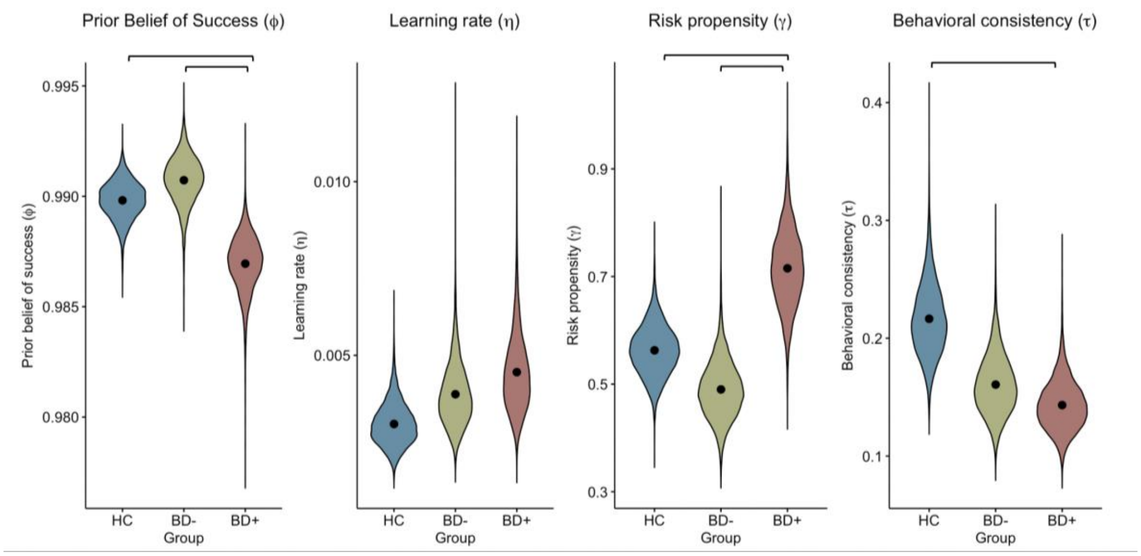

Note. Violin plots based on 8000 post-warmup MCMC samples. Horizontal bars indicate credible differences between two groups based on 90% HDI of posterior differences for the given parameter. BSR = Bayesian Sequential Risk-Taking Model (Park et al., 2021); BD+ = bipolar disorder (BD) with prior substance use disorder (SUD); BD- = BD without prior SUD; HC = healthy comparisons.

6.3. Correlations – BSR parameters and self-report/neuropsychological data: Correlations for the BSR model were similar to those found using the EWMV model (Supplement 4.2). To summarize: (1) poorer executive functioning performance correlated with higher learning rates (η) and lower behavioral consistency (τ); (2) lower BIS correlated with higher risk propensity (γ); and (3) greater susceptibility to boredom on the SSS was associated with higher risk propensity (γ), higher learning rates (η) and lower consistency (τ).

6.4. Further discussion: Others (Park et al., 2021) have shown increased BSR risk propensity estimates tend to manifest as reduced loss aversion (not increased risk preference) using EWMV model. This may represent a nuance of the EWMV model (e.g., loss aversion parameter may absorb variance related to motivation processes from the risk preference parameter, making it more sensitive to motivational differences).

7. Post-hoc analysis of adjusted pumps over time

7.1. Background: Based on results of analyses on EWMV parameters, BD groups (especially BD+) appeared to possess underlying vulnerabilities to risky behavior (i.e., reduced behavioral consistency; lower loss aversion in BD+ only), but did not show overall behavioral differences according in their adjusted average pumps on the task. We theorized that, in the case of BD+, these vulnerabilities may have been offset by their more pessimistic (more conservative) prior beliefs, which is why no differences were found in adjusted pumps. If this were the case, we would expect lower adjusted pumps in earlier blocks (due to more pessimistic prior beliefs) followed by increasing risk-taking (according to adjusted pumps) over the course of the task, facilitated by lower loss aversion and behavioral consistency. If change over time is observed, this may be the reason that higher learning rates were observed in BD+. In other words, they were updating more than HC from a conservative prior belief partially due to lower loss aversion. In the case of HC, where lower updating rates were observed (relative to BD+) and more consistent behavior was found relative to both BD groups, we would expect to see relatively few changes in adjusted pumps over time.

7.2. Methods: We divided the 20 trials of the BART into 3 blocks: block 1 (balloons 1 to 7), block 2 (balloons 8 to 14), and block 3 (balloons 15 to 20) and calculated adjusted pumps within each block. One HC was excluded from this analysis because they popped the balloon on every trial in block 3 and, consequently, an adjusted pumps score for block 3 could not be calculated. A mixed-model ANOVA with group (BD+, BD-, HC) as a between-subjects factor and block (1, 2, 3) as a within-subjects factor was performed. Additional post-hoc t-tests only examining BD+ and HC were performed for block 1 ($t = -1.94$, $p = 0.059$) and block 3 ($t = -0.02$, $p = 0.984$); neither showed significant differences.

7.3. Results: Adjusted pumps in each block in each of the three groups are illustrated in the figure below. Although the Block × Group interaction ($F = 2.32$, $p = 0.06$) did not reach statistical significance (perhaps due to low statistical power because of small sample sizes), the overall pattern aligned with our speculation that BD+ behaved more conservatively at the start of the task and increased their levels of risk-taking over the course of the task, which may have been driven, in part, by reduced loss aversion.

7.3.1. Adjusted pumps across three blocks of the BART

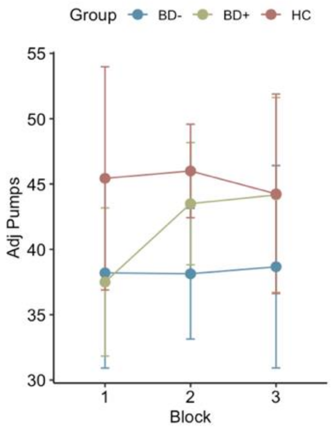

__Mixed ANOVA results__
Group: $F = 1.39$, $p = 0.257$
Block: $F = 1.44$, $p = 0.242$
Group x Block: $F = 2.32$, $p = 0.061$

Note. Error bars indicate the standard error. Block 1 = balloons 1-7; block 2 = balloons 8-14; block 3 = balloons 15-20. BD+ = bipolar disorder (BD) with prior substance use disorder (SUD); BD- = BD without prior SUD; HC = healthy comparisons. Post-hoc t-tests between BD+ and HC for block 1 ($t = -1.94$, $p = 0.059$) and block 3 ($t = -0.02$, $p = 0.984$) were also performed and neither showed significant differences

7.4. Assuming a flat burst probability. One reviewer asked why we assumed a flat burst probability in both the EWMV and BSR models when the participants were trained on a task with a variable range of balloon break points. One could argue that the probability of the balloon bursting should match this training phase (i.e., modeling a variable burst probability per trial rather than a constant one), but the use of a flat probability is well-supported by prior evidence. This choice is based on findings from Wallsten et al. (2005; the BSR development paper) and Pleskac (2008). First, Wallsten et al. (2005) evaluated several models assuming stationary (or 'flat') versus non-stationary burst probabilities. The winning model(s) used stationary values of p_k*_burst.* Models which included increasing values for p_k*_burst* performed poorly in comparison (see Wallsten et al. [2005], Table 2 and Table 3). Although an increasing p_k*_burst* is possible theoretically, these results indicate that this is not how participants behave. Next, Pleskac (2008) went even further, asking if the BSR model can tell the difference between when the probabilities underlying participants' behavior do/do not follow a flat distribution. They did this by arranging a task analogous to the BART and manipulating participants' probability expectations in different conditions. Results showed that the BSR model *can* tell the difference: when the underlying behavior is a stationary, stochastic process (flat), the flat model works better; when using a stochastic assumption where it is clearly not flat (nonstationary), the nonstationary model fit better. This means the BSR model is diagnostic and can be used to potentially detect differences in whether the burst probabilities underlying participants' behavior are flat or not. So, considering that the BSR model can be sensitive to the underlying burst probabilities AND that Wallsten et al. (2005) found convincing evidence for models that assumed a *flat* burst probability, this indicates that participants use a flat burst probability when making decisions on the BART. Therefore, we decided to maintain the flat probability of b p_k*_burst* in our models, which updates after each balloon but is fixed while pumping.

References

Ahn, W.-Y., Haines, N., & Zhang, L. (2017). Revealing Neurocomputational Mechanisms of Reinforcement Learning and Decision-Making With the hBayesDM Package. *Computational Psychiatry, 1*, 24–57.

Anderson, P., Anderson, V., & Lajoie, G. (1996). The Tower of London Test: Validation and standardization for pediatric populations. *Clinical Neuropsychologist, 10*(1), 54–65.

Carver, C. S., & White, T. L. (1994). Behavioral Inhibition, Behavioral Activation, and Affective Responses to Impending Reward and Punishment: The BIS/BAS Scales. *Journal of Personality and Social Psychology, 67*(2), 319–333.

Gray, J. A. (1981). *A critique of Eysenck's theory of personality*. In H. Eysenck (Ed.), A model for personality (pp. 246–276). Springer.

Park, H., Yang, J., Vassileva, J., & Ahn, W. Y. (2021). Development of a novel computational model for the Balloon Analogue Risk Task: The exponential-weight mean–variance model. *Journal of Mathematical Psychology, 102,* 102532.

Pickering, A. D., Corr, P. J., Powell, J. H., Kumari, V., Thornton, J. C., & Gray, J. A. (1997). Individual differences in reactions to reinforcing stimuli are neither black nor white: To what extent are they Gray? In H. Nyborg (Ed.), *The scientific study of human nature: Tribute to Hans J. Eysenck at eighty (*pp. 36–67). Pergamon/Elsevier Science.

Pleskac, T. (2008). Decision making and learning while taking sequential risks. *Journal of Experimental Psychology: Learning, Memory, and Cognition*, *34*(1), 167–185.

Reitan, R. M. (1958). Validity of the Trail Making Test as an Indicator of Organic Brain Damage. *Perceptual and Motor Skills, 8*, 271–276.

Rosen, W. G. (1980). Verbal fluency in aging and dementia. *Journal of Clinical and Experimental Neuropsychology*, *2(*2), 135–146.

Stroop, R. (1935). Studies of Interference in Serial Verbal Reactions. *Journal of Experimental Psychology*, *18*(6), 643.

Wallsten, T. S., Pleskac, T. J., & Lejuez, C. W. (2005). Modeling behavior in a clinically diagnostic sequential risk-taking task. *Psychological Review*, *112*(5), 862.

Wechsler, D. (2008). *Wechsler adult intelligence scale–Fourth Edition (WAIS-IV).* NCS Pearson.

Zuckerman, M., Kolin, E. A., Price, L., & Zoob, I. (1964). Development of a sensation-seeking scale. *Journal of Consulting Psychology*, *28*(6), 477–482.
